# The Rice *YL4* Gene Encoding a Ribosome Maturation Domain Protein Is Essential for Chloroplast Development

**DOI:** 10.3390/biology13080580

**Published:** 2024-07-31

**Authors:** Yunguang Sun, Yanxia Liu, Youze Zhang, Dongzhi Lin, Xiaobiao Pan, Yanjun Dong

**Affiliations:** 1 College of Life Sciences, Shanghai Normal University, Shanghai 200234, China; 15968503440@163.com (Y.S.); 1000526248@smail.shnu.edu.cn (Y.L.); 1000550094@smail.shnu.edu.cn (Y.Z.); dzlin@shnu.edu.cn (D.L.); 2 Shanghai Key Laboratory of Plant Molecular Sciences, Shanghai 200234, China; 3 Crop Institute, Taizhou Academy of Agricultural Sciences, Linhai 317000, China

**Keywords:** rice, map-based cloning, leaf color mutant, chloroplast development, CRM domain protein, *LOC_Os04g39060*

## Abstract

**Simple Summary:**

CRM domain proteins play key roles in plant growth and chloroplast development. To explore the role of CRM domain protein in the chloroplast development of rice, we cloned a rice CRM domain protein gene, *YL4*, and then conducted a tissue-specific expression analysis, subcellular localization, and transcription analysis. The experimental results showed that *YL4* was highly expressed in leaves and localized to the chloroplast, and its mutation affected chloroplast-related gene transcription levels and some agronomic traits. These results indicate that the *YL4* gene is a pivotal regulatory factor in chloroplast development and rice growth.

**Abstract:**

Chloroplast RNA splicing and ribosome maturation (CRM) domain proteins are a family of plant-specific proteins associated with RNA binding. In this study, we have conducted a detailed characterization of a novel rice CRM gene (*LOC_Os04g39060*) mutant, *yl4*, which showed yellow-green leaves at all the stages, had fewer tillers, and had a decreased plant height. Map-based cloning and CRISPR/Cas9 editing techniques all showed that *YL4* encoded a CRM domain protein in rice. In addition, subcellular localization revealed that YL4 was in chloroplasts. *YL4* transcripts were highly expressed in all leaves and undetectable in roots and stems, and the mutation of *YL4* affected the transcription of chloroplast-development-related genes. This study indicated that *YL4* is essential for chloroplast development and affects some agronomic traits.

## 1. Introduction

The chloroplast is the pivotal organelle in plants, serving as the primary site for photosynthesis. The chloroplast biogenesis is very complex and is coordinately regulated by both plastid and nuclear genes to complete the biosynthesis and assembly of functional chloroplasts. It has been established that plastid-encoded polymerase (PEP) and nuclear-encoded polymerase (NEP) play key roles in the regulation of chloroplast development [1,2]. The chloroplast genome encodes about 100 proteins, some of which include introns that cannot self-splice. The primary RNA transcription of these chloroplast genes requires ribozyme splicing, possibly through a chemical mechanism akin to nuclear splicing [2,3,4]. In plants, introns can be divided into groups I and II based on the conservation of their primary and secondary structures and the splicing mechanism of RNA splicing, and group II introns can be further classified into subgroups IIA and ⅡB [5,6]. Among them, chloroplast RNA splicing and ribosomal maturation (CRM) domain proteins are important players [7].

The CRM domain protein is a protein found in the plant-specific protein family that is related to RNA binding [8,9], which is associated with the metabolism of group I and II introns, as well as 23SrRNA, and they are the only known RNA ligands of them and exert a crucial regulatory influence in plants [10]. The CRM domain protein contains 14 homologous genes, with some members targeting chloroplasts and others mitochondria [4,11,12,13]. According to the quantification and architectural characteristics of the CRM domain, the CRM domain protein family is classified into four distinct subfamilies: CRS1 subfamily, CAF subfamily, subfamily 3, and subfamily 4 [11]. Among them, the first to three subfamilies have been documented to exert regulatory control over the splicing processes of both group I and II introns, while subfamily 4 is related to the assembly of large ribosomal subunits [4,14].

At present, the CRM domain proteins have been identified in *Arabidopsis*, maize, and rice and are essential for chloroplast development, gene expression regulation, and response to various abiotic stress. The first CRM domain protein, ZmCRS1, was identified and cloned in maize, which belongs to the CRS1 subfamily, contains three CRM domains, and can regulate the splicing of *atpF* intron in chloroplas [8,9]. *Arabidopsis* CFM9 belongs to subfamily 3 and contains one CRM domain that affects the splicing of seven introns in mitochondria [15]. In addition, CRM domain proteins are integral to ribosome development and response to abiotic stress, e.g., AtCFM4 belongs to the subfamily 4 and contains one CRM domain that is localized to chloroplasts, affects the assembly process of ribosome subunits 16S and 4.5S, and exhibits sensitivity to abiotic stress [14]. Additionally, CRM domain proteins are vital for plant growth and development, particularly in the splicing process of chloroplast gene introns [16].

In this research, we utilized a novel yellow-green leaf mutant, *yl4*, that exhibits a yellow-green leaf phenotype during the whole growth period. The mutation of rice *YL4*, encoding the CRM domain protein belonging to the CRS1 subfamily, was responsible for the mutant phenotype. In addition, the mutation of *YL4* led to delayed chloroplast development and affected transcript levels of genes involved in chloroplast development. These results indicated that *YL4* is essential for chloroplast development and has certain effects on some agronomic traits.

## 2. Materials and Methods

### 2.1. Plant Materials and Growth Conditions

The yellow-green leaf mutant *yl4* was initially identified from the M_2_ progeny of a *japonica* rice variety, Jiahua 1 (WT), induced by ^60^Co gamma-ray irradiation in 2006. After multiple rounds of self-pollination and selection, the mutant exhibited a stable and uniform phenotype along with consistent agronomic traits. The F_2_ population for genetic mapping was generated from a cross between Pei’ai 64S (*indica*) and the *yl4* mutant. The distinctive yellow-green leaf phenotype of the *yl4* mutant is discernible from the standard green leaf color under local cultivation conditions in both Hainan (winter season, subtropical climate) and Shanghai (spring season, temperate climate), China. In this study, seedlings of wild type (WT), the *yl4* mutant, and F_2_ generation were grown in controlled-environment growth chambers. The conditions included a photoperiod of 16 h of light and 8 h of darkness, with a light intensity of 120 μmol photons m^−2^ s^−1^ and a constant temperature of 32 °C.

### 2.2. Phenotype Observation and Photosynthetic Pigment Measurements

To elucidate the changes in leaf chlorophyll content and plant height during the whole vegetative growth period under natural conditions, leaf chlorophyll SPAD value and plant height were investigated weekly with rapid non-destructive chlorophyll meter (SPAD-502, Minolta, Osaka, Japan) and tape measure, respectively, for a total of nine weeks from the first week after transplanting [17,18]. Finally, the yield-associated characters (panicle number, panicle length, and 1000-grain weight) were investigated at maturity.

### 2.3. Transmission Electron Microscopy (TEM) Analysis

For elucidating the structure of chloroplasts using transmission electron microscopy, the uniform transverse leaf sections of the 3-leaf stage were harvested from the 3-leaf-stage *yl4* and WT seedlings. Then, the specimens were immersed in a mixture of 2.5% glutaraldehyde and 1% osmic acid in phosphate-buffered solution and preserved at a temperature of 4 °C in a centrifuge tube. After rinsing each with phosphate buffer, they were further dehydrated with a series of ethanol [starting at 50% and increasing to 100% (*v*/*v*)]. Subsequently, the samples were embedded in Spurr’s resin and heated to 60 °C. Finally, the samples were re-stained and examined under a transmission electron microscope (Hitachi-7650, Tokyo, Japan).

### 2.4. Map-Based Cloning of YL4

The rice genomic DNA was isolated from leaf tissues by the enhanced CTAB technique [19]. Initially, a set of 162 seeding with mutant phenotypes in F_2_ were randomly selected from the cross of Pei’ai 64S/*yl4* for preliminary linkage analysis; subsequently, the fine localization was carried out using 3852 seedings with mutant F_2_ plants. Novel InDel markers were designed with the PREMIRE5.0 software, leveraging the complete genomic data from the *japonica* variety Nipponbare [20,21] and the *indica* variety 9311 [22,23] (Supplemental Table S1). In addition, using such as TIGR (http://rice.plantbiology.msu.edu/cgi-bin/gbrowse/rice/, accessed on 5 January 2014) and KOME (http://cdna01.dna.affrc.go.jp/cDNA/index.html, accessed on 5 January 2014), respectively, to obtain candidate genes and full-length cDNA sequences. Then, all potential genes identified from the *yl4* mutant and WT plant were comprehensively amplified and sequenced, respectively.

### 2.5. Knockout of YL4

To verify whether *YL4* mutation caused the mutant phenotype, we used CRISPR/Cas9 genome editing technology to knockout *YL4* in WT plants. First, the Cas9 targeting construct of *YL4 (LOC_Os04g39060)* was generated using CRISPR Primer Designer website (https://crispr.dbcls.jp/, accessed on 20 October 2016). Then, the targeting sequence was amplified using the gene-specific primers (F: 5′-GCCGGATGCTTTGAAGCGTTCTG-3′; R: 5′-AAACCAGAACGCTTCAAAGCATC-3′) and inserted into the region between the OsU6a promoter and the gRNA scaffold. Finally, the resultant plasmids were introduced into Agrobacterium tumefaciens EHA105 and transformed into callus of WT plants by Agrobacterium tumefaciens-mediated transformation. Then, the obtained T_0_ and T_1_ transgenic plants were grown to investigate the phenotype.

### 2.6. Subcellular Localization of YL4

To understand the intracellular localization of YL4, a cDNA segment corresponding to the N-terminal domain (encompassing amino acids 1-178) of *YL4* was selectively amplified from total RNA extracted from WT plants. This amplification was achieved using a primer pair F: 5′-GAAGATCTATGCTCCTCCTCTTCCTCCC-3′ (*BglII*) and R: 5′-GGGGTACCGCCTCCAGTTTTT CTCTCG-3′ (*KpnI*) (the underlined sequences represent cleavage sites of *Bgl*II and *Kpn*I, respectively). The amplified fragment was seamlessly cloned into the pMON530-GFP vector, ensuring it was in frame with the GFP reporter. Then, the recombinant plasmid (pMON530-YL4-GFP) was under the control of the CaMV35S promoter. Subsequently, the tobacco (*Nicotiana tabacum*) leaves were subjected to the Agrobacterium (GV3101)-mediated transformation using the recombinant plasmid (pMON530-YL4-GFP). Meanwhile, a parallel transformation was performed using the pMON530-GFP vector devoid of the insert to serve as a negative control. The Agrobacterium suspension was delivered into the leaf tissue through the stomata using a 1 mL plastic syringe and gentle pressure to the lower epidermis. After two days at 25 °C, the GFP fluorescence in transgenic tobacco cells was examined using a Zeiss confocal laser scanning microscope (LSM 5 PASCAL, Oberkochen, Germany, http://www.zeiss.com, accessed on 15 April 2024) to ascertain the subcellular localization of the YL4-GFP fusion protein.

### 2.7. Sequence Alignment and Phylogenetic Analysis

The Rice Genome Annotation Project database served as a foundational reference for the prediction of genes. Using the Blast search tool, which is housed within the National Center for Biotechnology Information (NCBI, http://www.ncbi.nlm.nih.gov/, accessed on 15 April 2024), we identified homologous sequences of YL4 across various species. Subsequently, we performed multiple sequence alignments and constructed a phylogenetic tree employing the neighbor-joining tree method. This analysis utilized MEGA v6.06 software, which incorporated a bootstrap test consisting of 1000 replicates [24]. Furthermore, the sequences of several of the most similar homologs were aligned using DNAMAN 9.0.

### 2.8. RNA Extraction, RT-PCR, and Quantitative Real-Time PCR

Total RNA was isolated from various tissues of the WT seedlings, encompassing the roots, stems, and leaves, as well as from specific structures such as flag leaves, second leaves, and young panicles during the heading stage. The extraction was facilitated by the TRIzol Reagent (Invitrogen, Waltham, MA, USA; http://www.invitrogen.com, accessed on 15 April 2024) and subsequently treated with DNaseI by an RNeasy kit (Qiagen, Dusseldorf, Germany; http://www.qiagen.com, accessed on 15 April 2024) method following the manufacturer’s instructions. The first-strand cDNA was executed utilizing the Revert-Aid first-strand cDNA synthesis kit (Toyobo, Osaka, Japan; http://www.toyobo.co.jp, accessed on 15 April 2024). RT-PCR analysis was carried out to assess the transcription levels of tested genes.

For transcriptional analysis of the genes implicated in Chl biosynthesis and chloroplast development and photosynthesis in *yl4* mutant, 24 genes (*CAO1*, *PORA*, *YGL1*, *Cab1R*, *Cab2R*, *RbcS*, *RbcL*, *PsaA*, *PsbA*, *LhcpⅡ*, *OsRpoTp*, *rps7*, *V1*, *V2*, *RNRL*, *RNRS*, *RpoB*, *16SrRNA*, *23SrRNA*, *Rps20*, *FtsZ*, *Rpl21*, *OsDG2*, and *YL4*) were curated and selected. Quantitative real-time PCR amplification was conducted on the Bio-Rad IQ5 Real-Time system with the SYBR Green Mix (Takara, Osaka, Japan). Each sample was performed in triplicate to ensure the accuracy and reliability of the qRT-PCR data. *OsActin*, a housekeeping gene, served as the internal control for normalization. The primer sequences for qRT-PCR are detailed in Supplemental Table S2. The relative quantification of gene expression was determined using the 2^−ΔΔCT^ method [25]. In addition, the 2^−ΔCT^ method meant that ΔCT represents the difference in CT values between the target genes and the *OsActin*. Throughout this investigation, the data were means ± SD (n = 3).

## 3. Results

### 3.1. Characterization of the yl4 Mutant

**Figure 1:**
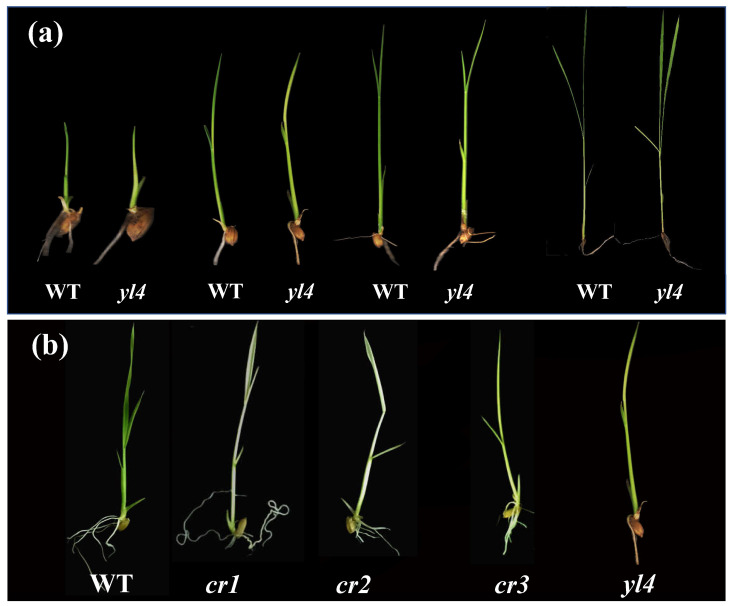
Phenotypic observation of the *yl4* mutant: (**a**) one-, two-, three- and four-leaf-stage seedlings of wild type (WT) and *yl4* mutant; (**b**) comparison of WT plants, *yl4* mutant, and gene-edited seedlings (*cr1–3*).

**Figure 2:**
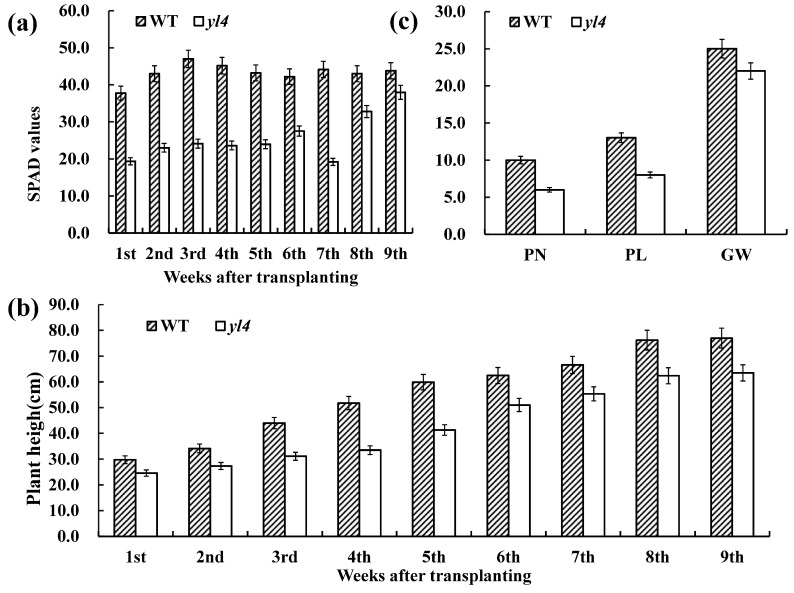
Characterization of the *yl4* mutants: (**a**) leaf chlorophyll SPAD during the whole vegetative growth period after transplanting; (**b**) plant height during the whole vegetative growth period after transplanting; (**c**) panicle-related traits between *yl4* mutant and WT plants. PN, panicle number; PL, panicle length (cm); GW, 1000-grain weight (g). Error bars represent SD (n = 3).

It was observed that the *yl4* mutant showed a yellow-green leaf phenotype during the whole growth period (Figure 1a). In addition, the leaf chlorophyll SPAD values and plant height in *yl4* plants were drastically lower than WT plants after transplanting under natural conditions, and the plant height, grain weight (GW), panicle number (PN), and panicle length (PL) at maturity were significantly reduced (Figure 2).

**Figure 3:**
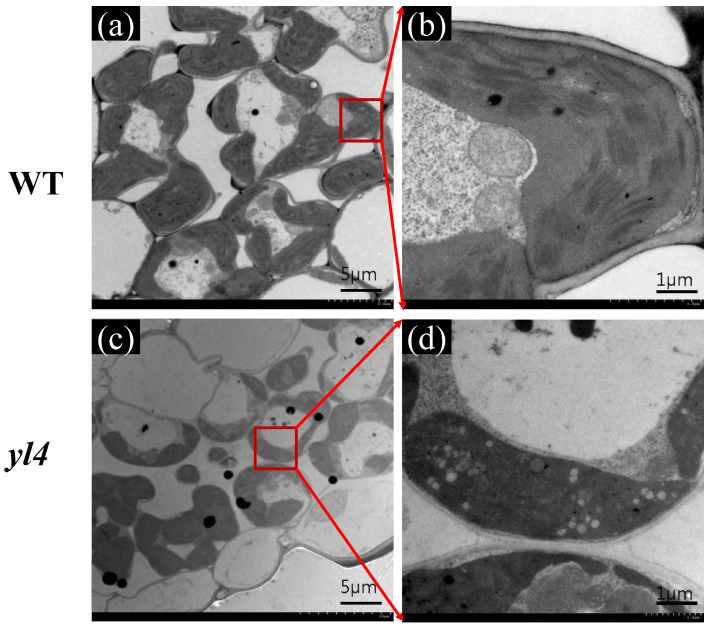
Transmission electron microscopic images of chloroplasts in WT and *yl4* mutant of the 3-leaf stage: (**a**,**b**) chloroplast structure in WT; (**c**,**d**) chloroplast structure in *yl4.* The red arrow indicates the enlarged image in the red box.

To ascertain whether the observed deficiency in photosynthetic pigments in the *yl4* mutant was concomitant with alterations at the ultrastructural level within the chloroplasts, we investigated the ultrastructure of chloroplast (Figure 3). As a result, *yl4* cells had fewer chloroplasts than WT cells, with vacuolated and lacked organized lamellar. Thus, it was presumed that the abnormal chloroplast may adversely impact the chlorophyll content in the mutant.

### 3.2. Map-Based Cloning of YL4

**Figure 4:**
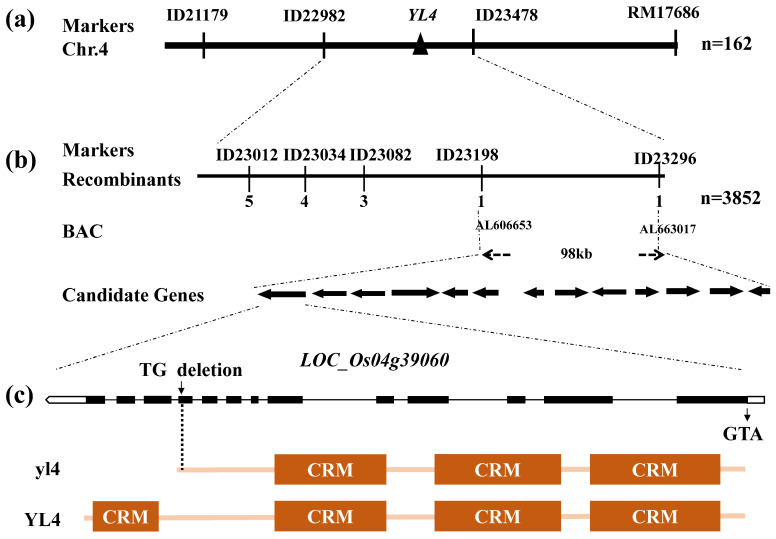
Map-based cloning of *YL4*: (**a**) the *YL4* gene was initially located on chromosome 4; (**b**) *YL4* was narrowed to 98 kb; (**c**) location of the deletion (TG) and CRM domains in *yl4* mutants.

All F_1_ plants derived from the hybridization of the *yl4* with Pei’ai 64S displayed a normal green color indicative of the phenotype of recessive mutation of the *yl4*. In the subsequent F_2_ generation, the mutant phenotype was segregated as a monogenic recessive Mendelian trait (Supplemental Table S3, χ^2^ = 2.90 < χ^2^_0.05_ = 3.84). Subsequently, we initiated the genetic mapping process by selecting 162 F2 individuals exhibiting the mutant phenotype. This effort led to the position of the *YL4* locus between markers ID21179 and RM17686 on chromosome 4 (Figure 4a). Lastly, we refined the mapping of the *YL4* locus to a precise 98 kb genomic segment between markers ID23198 and ID23296 within the BAC clones AL606653 and AL663017 utilizing 3852 F_2_ mutant individuals (Figure 4b). This interval contained 13 candidate genes (Figure 4b). Through sequencing of these candidate genes, a singular mutation was identified as 2bp (TG) deletion at 2461bp of the ATG start codon within the 10th exon of *LOC_Os04g39060* (Figure 4c).

To further verify whether *LOC_Os04g39060* represented the *YL4* gene, we used the CRISPR/Cas9 genome editing system to knockout *LOC_Os04g39060* in WT plants. As a result, three kinds of homozygous T_1_-edited plants (Figure 1b) were obtained, carrying three different editing sites (the 3bp (AAG) deletion at position 2069 bp in *cr3* led to K (Lysine) deletion at 690aa; both the 1bp(T) in *cr1* and 1bp(A) in *cr2* insertion at the position 2071 bp from of the ATG start codon in *YL4* led to early termination, respectively). Importantly, all homozygous edited plants showed similar phenotypes to *yl4* mutants (Figure 1b). Taken together, these results confirmed that *LOC_Os04g39060* was indeed the *YL4* gene.

### 3.3. Characterization of YL4 Protein

**Figure 5:**
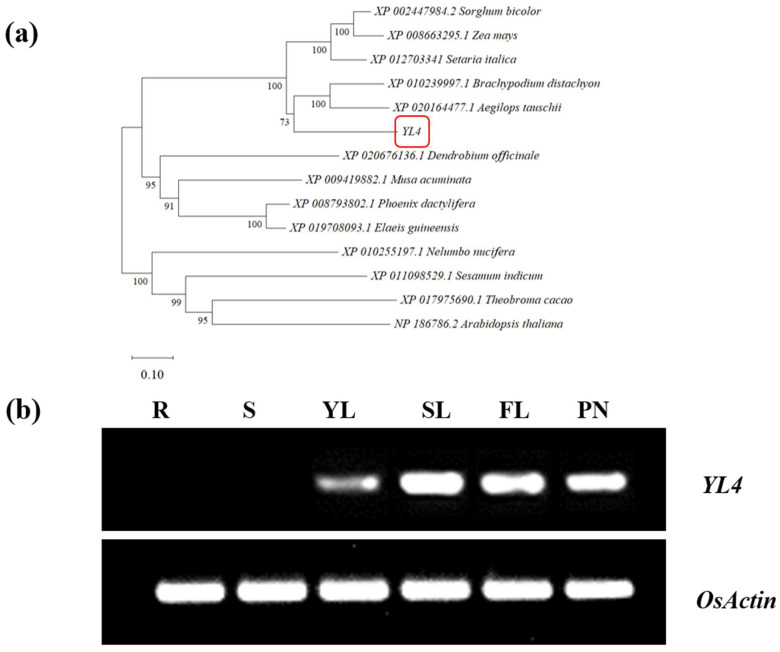
Phylogenic tree of YL4 protein and expression pattern of *YL4*: (**a**) phylogenic tree of YL4 and homologs. The red box represents YL4; (**b**) expression pattern of *YL4*. R, root; S, stem; YL, young leaf; SL, second leaf; FL, flag leaf; PN, panicle.

The *YL4* gene consists of 13 exons and 12 introns and encodes the CRM protein with 1013aa polypeptide, containing four CRM domains, which was classified as CRS1 subfamily [11] (Figure 4c) (http://rice.plantbiology.msu.edu, accessed on 15 April 2024). Further, it was found that the *yl4* mutant lost the fourth CRM domain (Figure 4c). In addition, BLAST searches of the available genome sequences with the YL4 revealed close homologs in *Brachypodium distachyon*, *Aegilops tauschii*, *Setaria italica*, *Sorghum bicolor*, *Zea mays*, and *Phoenix dactylifera*, indicating that YL4 exhibits a high degree of conservation within higher plants (Supplemental Figure S1). Furthermore, the phylogenetic examination delineated a clear bifurcation of YL4 homologs into two categories: monocots and dicotyledons. This classification is in concordance with the established biological taxonomy (Figure 5a).

### 3.4. Expression Pattern and Subcellular Localization of YL4

**Figure 6:**
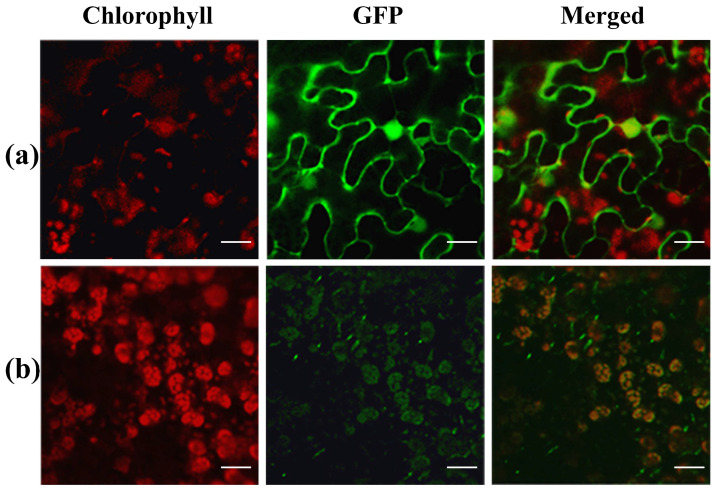
Subcellular localization of YL4 protein: (**a**) empty GFP vector without a specific targeting sequence; (**b**) YL4-GFP fusion. The scale bar represents 20 μm.

According to the Rice Annotation Project Database (https://rapdb.dna.affrc.go.jp/, accessed on 15 April 2024), *YL4* is mainly expressed in leaves (Supplemental Figure S2). To confirm the expression patterns of the *YL4* gene, we analyzed the *YL4* transcript level of the roots, stems, young leaves, second leaves, flag leaves, and panicles in WT plants. As shown in Figure 5b, *YL4* was abundantly expressed in leaves and panicles but hardly in roots and stems, which was basically consistent with the predicted results (Supplemental Figure S2). In addition, it is predicted that YL4 protein had a high probability of locating in chloroplasts (http://www.cbs.dtu.dk/services/TargetP/, accessed on 15 April 2024). To elucidate the precise subcellular localization of YL4 protein, a recombinant vector, pMON530-YL4-GFP, was constructed and then introduced into tobacco cells on a transient through Agrobacterium-mediated transformation. As predicted, almost all of the green fluorescent signal from the YL4-GFP fusion protein was found to overlap with the chlorophyll autofluorescence in tobacco cells (Figure 6). It was clearly shown that YL4 was localized to the chloroplast.

### 3.5. The Transcript Expression of Related Genes in the yl4 Mutants

**Figure 7:**
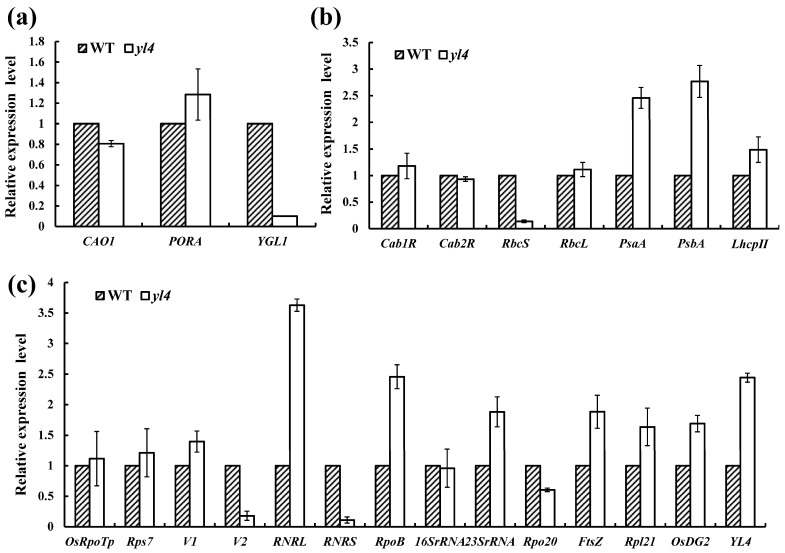
Quantitative expression analysis of genes associated with chlorophyll biosynthesis (**a**), photosynthesis (**b**), and chloroplast development (**c**) in WT and *yl4* mutant. The expression level of each gene was analyzed by qRT-PCR and *OsActin* as a control. Error bars represent SD (n = 3).

To determine the impact of the *YL4* mutation on the transcription levels of genes for Chl biosynthesis, photosynthesis, and chloroplast development, we investigated the transcription levels of 23 known related genes and *YL4* (Figure 7). Resultantly, an obvious decrease in *YGL1* was observed; however, there was a slight change in Chl synthesis *CAO1* [26] and *PORA* [27] in the *yl4* mutant (Figure 7a). Among the seven photosynthesis-related genes (Figure 7b), *RbcS* [28] were severely inhibited, and *PsaA* and *PsbA* [29] were all significantly up-regulated in the *yl4* mutant. Moreover, with regard to the expression of chloroplast development-associated genes, *V1* [30], *RNRL*(*V3*) [31], *RpoB* [32], *23SrRNA* [33], *FtsZ* [34], and *RPL21* were obviously up-regulated, while *V2* [35] and *RNRS* were considerably down-regulated (Figure 7c). In addition, we found that the expression level of *YL4* was significantly up-regulated in the *yl4* mutant. In conclusion, the altered expression patterns of chlorophyll biosynthesis and chloroplast biogenesis genes may contribute to the yellow-leaf phenotype in the *yl4* mutant.

## 4. Discussion

In the present investigation, we delineated the phenotype and genetic basis of the yellow-leaf mutant *yl4*, which is distinguished by a complete deficiency of chlorophyll and aberrant chloroplast morphology during the whole growth period. Through a map-based cloning approach, we successfully cloned the *YL4* gene, discovering that it encodes a chloroplast-localized CRM protein with four CRM domains. The 2bp (TG) deletion of the 10th exon within the last CRM domain resulted in a yellow-leaf phenotype. This mutation profoundly perturbed the expression levels of genes integral to chlorophyll biosynthesis, photosynthesis, and chloroplast development. In general, the changes in gene transcription levels also affect the expression of corresponding proteins. Since the mutation of the *YL4* gene has led to changes in the transcription levels of some genes related to chlorophyll biosynthesis, photosynthesis, and chlorophyll development (Figure 7), we can speculate that the expression of corresponding proteins should be affected. Of course, it is necessary to further ascertain the corresponding protein expressions between mutant and WT plants by Western blot analysis. Collectively, this study underscores the pivotal role of the rice *YL4* gene in orchestrating chloroplast maturation and function.

### 4.1. YL4 Acts during the First Step of Chloroplast Development

The chloroplast, an organelle with semi-autonomy, harbors approximately 100 genes, although it operates with over 3000 proteins [36]. As described previously, the maturation of plastids from proplastids to fully developed chloroplasts can be broadly categorized into three phases [37]: (i) the replication of plastids and the synthesis of plastid DNA; (ii) the formation of the plastid genetic machinery; (iii) the initiation of the photosynthetic apparatus. To date, the expressions of *RNRL*, *OsDG2*, and *FtsZ* have been identified as participating in the first phase. *V2*, *rpoB*, and *23SrRNA* are implicated in modulating chloroplast transcription/translation processes and are highly transcribed in the second phase. And *RbcS*, *PsaA*, and *PsbA*, all of which function in the regulation of photosynthetic apparatus, are involved in the third step. In this study, the *YL4* mutation all severely affected the expression of genes involved in the first (*RNRL*, *OsDG2*, and *FtsZ*), second (*V2, RpoB, 23SrRNA*), and third (*RbcS*, *PsaA*, *PsbA*) steps of chloroplast development (Figure 7). Thus, it is plausible to infer that *YL4* contributes to the development of chloroplasts, potentially regulating the first phase and subsequently influencing the subsequent phases in a cascading manner.

### 4.2. Multiple Functions of YL4 in Chloroplast Development

The previous findings underscore the pivotal roles of CRM domain-containing proteins in the realm of plant growth and development [11]. Mutations within genes that encode proteins with multiple CRM domains are associated with the manifestation of aberrant phenotypes and impeded developmental progression [14]. In the previous research, two allelic mutants, *oscfm2-1* and *oscfm2-2*, were obtained by gene editing in rice, which had 2 and 11bp deletions both happened in the first exon in *LOC_Os04g39060*, respectively, resulting in premature termination and the loss of all four CRM domains and led to a severe albino seedling [13]. However, the *YL4* mutation that happened in the 10th exon within the last CRM domain caused the yellow-green leaf phenotype and did not cause the severe albino phenotype. Compared with *oscfm2* mutants, *yl4* is a weak allelic mutant and still keeps the first three CRM domains functioning normally. Further, it was reported that the intron splicing of *ycf3-1*, *rpl2*, *rps12*, *atpF*, *ndhA*, and *trnL* in the *oscfm2* mutant is abnormal [13]. In view of these facts, we could conclude that *YL4* may have multiple functions in chloroplast development.

## 5. Conclusions

At present, there are few reports on the role of CRM domain proteins in rice, so it is urgent to further explore the mechanism of CRM domain proteins regulating chloroplast development and function in rice. In the study, we report a CRM domain protein YL4, whose mutation leads to abnormal chloroplast development in rice and has adverse effects on certain agronomic traits. The results indicate that YL4 plays an important role in the development of rice chloroplasts.

## Data Availability

The data presented in this study are available upon request.

## Supplementary Materials

The following supporting information can be downloaded at https://www.mdpi.com/article/10.3390/biology13080580/s1. Table S1. The PCR-based molecular markers designed for fine mapping; Table S2. Markers designed for real-time RT-PCR and gene function; Table S3. Genetic segregation analysis of *yl4* mutants in the F2 population; Figure S1. Multiple-sequence alignment of YL4 homologous proteins analyzed by DNAMAN and CRM domains of YL4; Figure S2. Website prediction results of *YL4*.
