# Supplementary material for: The Rice YL4 Gene Encoding a Ribosome Maturation Domain Protein Is Essential for Chloroplast Development
Source: Biology (Basel). 2024 Jul 31;13(8):580. doi: 10.3390/biology13080580 (PMC11351240; doi:10.3390/biology13080580)

Supplemental Table S1. The PCR-based molecular markers designed for fine mapping

No	Markers	Forward sequence (5' to 3')	Reverse sequence (5' to 3')
1	ID21179	ACTCGTTCTTGTAACCCGTTGG	ACAGACAGCCAAGCCTACCAA
2	ID22982	TTTCAACGAGCAAACCAACAACC	AACTCACTTCATGCGGTATCCT
3	ID23478	GAGGCTTTTCTTTCCTTTTC	AGGCAACGTGTCCCTTTC
4	RM17686	GAACGAAGTGAACAAGCCAATCC	CCCATTACGGCTTAGGCTCAGG
5	ID23012	GGTGAATCCATCGTGAGTGTGCTA	CCTTCAATCCTCCTACTCTTCCTC
6	ID23034	GCTCTATGACCGCAACTCTT	TTGCGTCTACGATGGTGT
7	ID23082	AGAGTTGAGTTGTAGAGTTGGGTCC	AGAAGAGTTACATGTTCCGCGTGG
8	ID23198	TGTTACGCTACTGAAGCAAGTGACG	CTTCTTTGCTCTGCTTGGTGTGC
9	ID23296	CGCTCCGTATCTGGATTCTG	CCTGTGTCCTACGCACCATA

Supplemental Table S2. Markers designed for realtime RT-PCR and gene function

Genes	Primer sequences (5’ to 3’)	Gene functions
CAO1	GATCCATACCCGATCGACAT<br>CGAGAGACATCCGGTAGAGC	Encoding chlorophyllide a oxygenase1
PORA	TGTACTGGAGCTGGAACAACAA<br>GAGCACAGCAAAATCCTAGACG	Encoding NADPH-dependent protochlorophyllide oxidoreductase
YGL1	CAGTCTCCAATGGCCACCT<br>TGCTTTCATCAGTGGCTGGT	Encoding a Chl synthetase
Cab1R	AGATGGGTTTAGTGCGACGAG<br>TTTGGGATCGAGGGAGTATTT	Encoding light-harvesting Chl a/b binding protein 1
Cab2R	AAAGGACATAACCTTGCAAG<br>AGTTTTCCTATTGAACCGTG	Encoding a component of the plastid division machinery
RbcS	TCCGCTGAGTTTTGGCTATTT<br>GGACTTGAGCCCTGGAAGG	Encoding the small subunit of Rubisco
RbcL	CTTGGCAGCATTCCGAGTAA<br>ACAACGGGCTCGATGTGATA	Encoding the large subunit of Rubisco
PsaA	GCGAGCAAATAAAACACCTTTC<br>GTACCAGCTTAACGTGGGGAG	Encoding the P700 chlorophyll a apoprotein of PS I
PsbA	CCCTCATTAGCAGATTCGTTTT<br>ATGATTGTATTCCAGGCAGAGC	Encoding the D1 protein of PS II
Lhcp II	GAAGAAGATCAAGAACGGCC<br>TTGCCGGGGACGAAGTTGGT	Encoding light-harvesting complex protein in PS II
OsRpoTp	AAGCAGACAGTGATGACATC<br>ATCACATGCATGCACCCAAA	Encoding RNA polymerase subunit of PEP
Rps7	GCCAAAATCCATTCCAATTC<br>GGAGATGTACACGAGGAGATTG	Encoding the small subunits ribosomal protein S7
V1	TCAGAACGAGAAGGATTCAGCA<br>GGCAACAGCCACTAAAATTTCT	Encoding a chloroplast localized protein NUS1
V2	GAGGAGTTCCTCACGATGAT<br>AGCATCAATGATAGACTCC	Encoding plastid/mitochondrial guanylate kinase (pt/mt GK)
RNRL	GTTAGATGCTTCACTACACAG<br>GTACCATTGCCAACATGGCAAC	Encoding the large subunits of ribonucleotide reductase
RNRS	GCCAAAATCCATTCCAATTC<br>GGAGATGTACACGAGGAGATTG	Encoding the small subunits of ribonucleotide reductase
RpoB	TTTGGTTTCGATGTGCA<br>TATGGTCTAATTCCGAGCGGT	Encoding RNA polymerase β subunits of PEP
16SrRNA	CCGTTGGTGTTCTTTCCGAT<br>TTCAAGTCCGCCGTCAAATC	Encoding chloroplast ribosomes small subunits of 16S components
23SrRNA	TGTGGGCGTTAGAGCATTGAG<br>CACTTGGCTACCCAGCGTTTA	Encoding chloroplast ribosomes large subunits of 23S components
rps20	CACGCTCTTCTCCCTCTCCT'<br>GTAGGAGGCGGACAGGCG	Encoding the small subunits ribosomal protein S20

FtsZ	AAAGGACATAACCTTGCAAG<br>AGTTTTCCTATTGAACCGTG	Encoding a component of the plastid division machinery
Rpl21	AAGAAGAGGAGGCTGCGGT<br>GACATTGGCGCCTTTCAGC	Encoding the large subunits ribosomal protein L21
OsDG2	AAACCGAAATCGTCGTGGAG<br>ACAAGGGAGCACCTGAACTAAGA	Glycine-rich protein (GRP), Regulation of chloroplast development at early leaf stage
OsActin	AGGAAGGCTGGAAGAGGACC<br>CGGGAAATTGTGAGGGACAT	As the internal control

Supplemental Table S3. Genetic segregation analysis of *yl4* mutants in the F_2 population

Cross	Observed number of F_2 plants			χ^2(3:1)	*P*
	Total	Green	Albino		
Pei'ai 64S/*yl4*	336	91	245	2.90＜3.84	0.07＞0.05

$\chi^2_{0.05}$=3.84

Supplemental Figure S1. Multiple-sequence alignment of YL4 homologous proteins analyzed by DNAMAN and CRM domains of YL4.

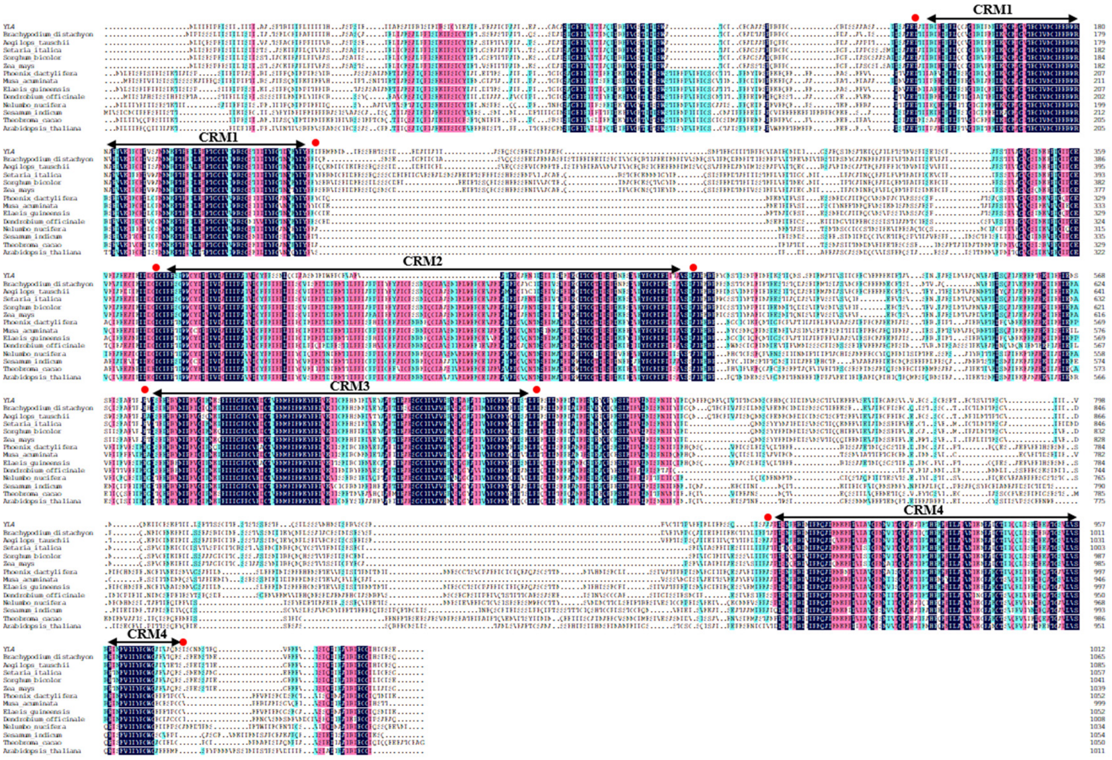

Supplemental Figure S2. Website prediction results of *YL4*

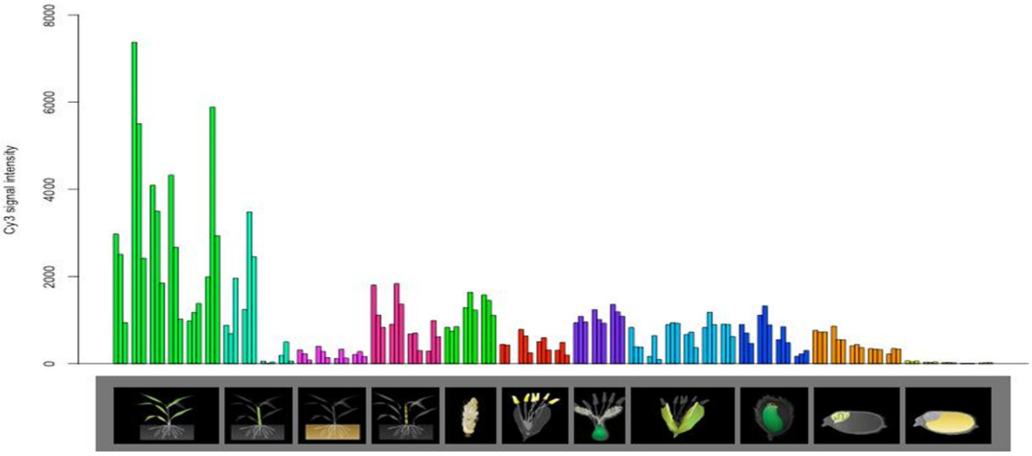

Supplement: Supplementary file 1 [file biology-13-00580-s001.zip › biology-3049453-supplementary.pdf]
